# Whole Exome Sequencing as a First-Line Molecular Genetic Test in Developmental and Epileptic Encephalopathies

**DOI:** 10.3390/ijms25021146

**Published:** 2024-01-17

**Authors:** Luigi Vetri, Francesco Calì, Salvatore Saccone, Mirella Vinci, Natalia Valeria Chiavetta, Marco Carotenuto, Michele Roccella, Carola Costanza, Maurizio Elia

**Affiliations:** 1 Oasi Research Institute-IRCCS, 94018 Troina, Italy; lvetri@oasi.en.it (L.V.); mvinci@oasi.en.it (M.V.); vchiavetta@oasi.en.it (N.V.C.); melia@oasi.en.it (M.E.); 2 Department Biological, Geological and Environmental Sciences, University of Catania, Via Androne 81, 95124 Catania, Italy; 3 Clinic of Child and Adolescent Neuropsychiatry, Department of Mental Health, Physical and Preventive Medicine, University of Campania “Luigi Vanvitelli”, 80131 Naples, Italy; marco.carotenuto@unicampania.it; 4 Department of Psychology, Educational Science and Human Movement, University of Palermo, 90141 Palermo, Italy; michele.roccella@unipa.it (M.R.); carola.costanza@unipa.it (C.C.)

**Keywords:** developmental and epileptic encephalopathy, whole-exome sequencing, next-generation sequencing, NGS, WES, DEE, epilepsy, seizure, intellectual disability, genetic diagnosis

## Abstract

Developmental and epileptic encephalopathies (DEE) are severe neurodevelopmental disorders characterized by recurrent, usually early-onset, epileptic seizures accompanied by developmental impairment often related to both underlying genetic etiology and abnormal epileptiform activity. Today, next-generation sequencing technologies (NGS) allow us to sequence large portions of DNA quickly and with low costs. The aim of this study is to evaluate the use of whole-exome sequencing (WES) as a first-line molecular genetic test in a sample of subjects with DEEs characterized by early-onset drug-resistant epilepsies, associated with global developmental delay and/or intellectual disability (ID). We performed 82 WESs, identifying 35 pathogenic variants with a detection rate of 43%. The identified variants were highlighted on 29 different genes including, 3 new candidate genes (*KCNC2*, *STXBP6*, *DHRS9*) for DEEs never identified before. In total, 23 out of 35 (66%) de novo variants were identified. The most frequently identified type of inheritance was autosomal dominant de novo (60%) followed by autosomal recessive in homozygosity (17%) and heterozygosity (11%), autosomal dominant inherited from parental mosaicism (6%) and X-linked dominant de novo (6%). The most frequent mutations identified were missense (75%) followed by frameshift deletions (16%), frameshift duplications (5%), and splicing mutations (3%). Considering the results obtained in the present study we support the use of WES as a form of first-line molecular genetic testing in DEEs.

## 1. Introduction

In 2022, the ILAE (International League Against Epilepsy) Task Force on Nosology and Definitions divided epileptic syndromes with onset in neonates and infants into two groups: self-limited epilepsy syndromes presenting with an age-related spontaneous remission and developmental and epileptic encephalopathies (DEEs), syndromes where there is a constant neurodevelopment impairment [1].

DEEs are severe neurodevelopmental disorders with a typically sporadic nature, characterized by recurrent epileptic seizures with onset in neonates or childhood accompanied by psychomotor delay and intellectual disability (ID) [2]. At the beginning, seizures are mostly drug-resistant and cognitive disorders follow their onset, which may subsequently attenuate over the course of the child’s development. In the course of the disease, various neurological signs may also arise, such as ataxia, movement disorders, and behavioral disorders [3]. Neuroimaging tests may not show specific structural abnormalities in the brains of patients with DEEs [4]. The typical course of DEEs has suggested that seizures and intercritical EEG abnormalities may contribute, at least in the early stages, to altering the normal cognitive development of the individual [5]. In this context, epileptic encephalopathies have been defined by the ILAE as “the condition in which epileptic activity itself contributes to severe cognitive and behavioral impairments above and beyond what might be expected from the underlying pathology” [6].

Attributing a direct pathogenic effect to the abnormal electrical activity rather than to the underlying genetic variation or to the acquired damage suffered is sometimes extremely complex. In many DEEs, the developmental disorder is not only due to the frequent epileptic activity, but mainly to the direct effect of the genetic mutation. These observations have led to the expansion of the terminology used and the inclusion of the term “developmental” in order to emphasize that both aspects (genetic cause and epilepsy) play an important role in the clinical presentation [7]. Therefore, in 2017 the ILAE suggested the use of the term “developmental and epileptic encephalopathy” (DEE) to emphasize that both aspects, genetics and electrical activity, can play a role in the clinical presentation [8].

Based on existing knowledge, hundreds of genes are associated with DEEs. The phenotypic spectrum of DEEs is, therefore, extremely broad, and includes a multitude of neurological signs, which are almost invariably associated with psychiatric manifestations and psychological and behavioral abnormalities [9,10,11].

Today, there are several genetic investigations available with different detection capabilities that can clarify the genetic etiology of DEEs. A possible flowchart, proposed by the Italian League Against Epilepsy (LICE) in 2016 for the genetic diagnosis of DEEs, distinguishes through phenotypic and electroclinical characterization suspected genetic epilepsies into defined or undefined epileptic syndromes. In the first case, a next-generation sequencing (NGS) panel of genes related to known epileptic syndromes is usually performed; subsequently, the possible presence of chromosomal rearrangements is searched through array-CGH, and finally, whole-exome sequencing (WES) is advised. In the second case, that is, in undefined epilepsies, if an association with ID, autism or dysmorphic features arises, the search for chromosomal rearrangements is carried out in the first instance through array-CGH, followed by the study of the karyotype for complex chromosomal rearrangements, and only at a later time is an NGS panel is performed. Finally, if all tests are negative, the WES is performed [12].

NGS is a technology used to detect the order of nucleotides in whole or targeted regions of DNA or RNA. It is a massively parallel sequencing technology characterized by ultra-high throughput, scalability, and speed. NGS technologies marked the beginning of the golden age for genetics, allowing large portions of DNA to be sequenced quickly and with reduced costs [13,14]. The impact of efficient and low-cost human exome sequencing has been amplified by the growing knowledge of the genetic background of different populations and the development of increasingly detailed maps of human polymorphisms.

The aim of the present study is to use exome sequencing as a first-line molecular genetic test in a sample of subjects with DEEs characterized by early-onset drug-resistant epilepsies, associated with global developmental delay and/or intellectual disability. WES, used as a first-level molecular genetic test, was applied to the family trio (affected patient and parents) in a cohort of patients referred to the Oasi Research Institute—IRCCS in Troina (Italy).

## 2. Results

As per the inclusion criteria, all study participants presented with epilepsy and cognitive impairment ranging from borderline to profound. Ninety-four patients with early-onset drug-resistant epilepsy with intellectual developmental impairment were recruited. Of these 94 subjects, 12 were excluded because they did not fully meet the inclusion/exclusion criteria: 7 cases had multi-organ abnormalities and facial dysmorphisms and were sent for traditional molecular cytogenetics, in 4 patients DNA from a parent could not be found, and finally the parents of 1 patient did not provide consent for the study.

**Figure 1:**
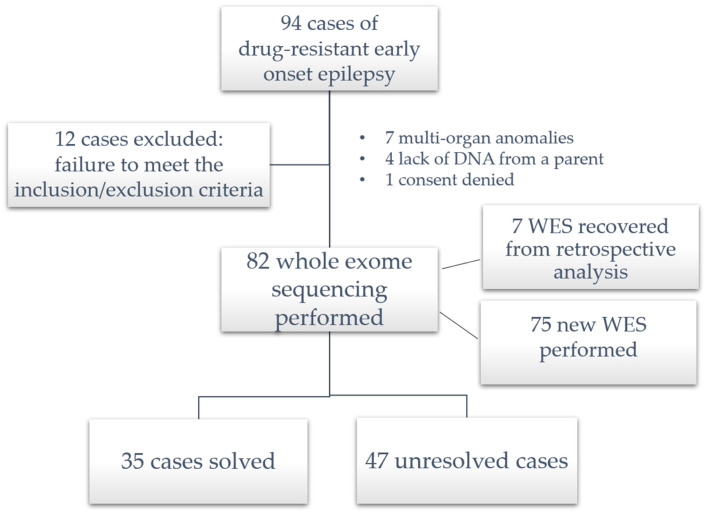
Enrollment of the study sample.

Therefore, the present study considered the exome sequencing of 82 subjects (the total number of exomes performed was limited by the emergency caused by the COVID-19 pandemic); 7 WESs were performed prior to study approval and were considered retrospectively as meeting the inclusion/exclusion criteria after retrieving the appropriate informed consent for data use (Figure 1).

### 2.1. Clinical Features

The study was carried out on 82 subjects with early-onset drug-resistant epilepsy and cognitive developmental impairment. Among them, 40 (49%) were males and 42 (51%) were females; the mean age at the time of testing was 10 years, while seizure onset was within 36 months with a mean of 16 months; neonatal onset (first four weeks of an infant’s life) was evidenced in 21% of cases, while the remaining 79% of cases had an infantile seizure onset, and a family history of epilepsy was present in 37% of cases. Focal abnormalities were the most frequently observed electroencephalographic changes (44%) followed by generalized abnormalities (18%) and multifocal abnormalities (17%). In 52% of cases, brain MRI showed no notable alterations; the most frequently described abnormal findings were brain atrophy (29%), ventricular dilatation (28%), and corpus callosum thinning/agenesis (23%). The number of antiepileptic drugs used before genetic testing ranged from 2 to 7.

The main characteristics related to the whole sample of the study are summarized in Table 1, while Table 2 summarizes, in more detail, the electroclinical and imaging characteristics of the 35 subjects with mutation detected by WES.

### 2.2. Genetic Findings

In total, 72 out of the 82 exome sequencings performed on the study sample were carried out on familial trios (father–mother–proband), while 5 were carried out in quarter mode (two probands, mother and father).

From the exome sequencing (trios or quadro) performed on 82 cases, 35 pathogenic or likely pathogenic variants compatible with the highlighted phenotype were identified. Thus, the detection rate of exome sequencing in this study was 43%. The identified variants were highlighted on 29 different genes, including 3 new candidate genes (~10%) for DEEs never identified before the present study. The de novo variants identified numbered 23 out of 35 (~66%). Variants with their pathogenic role identified are shown in Table 3.

**Figure 2:**
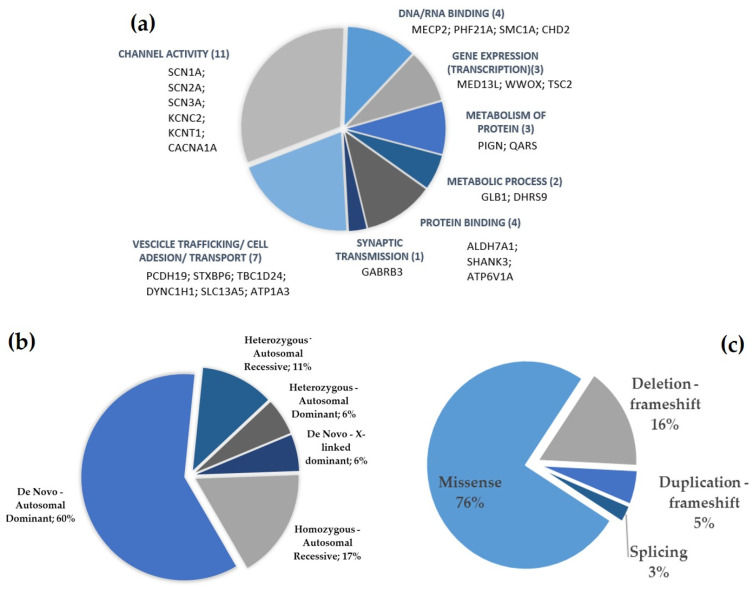
(**a**) Identified genes divided into macro-categories based on Gene Ontology (GO) or on pathway information. (**b**) Inheritance patterns of the variants identified. (**c**) Types of mutations identified.

The genes divided into macro-categories based on Gene Ontology (GO) or on pathway information are summarized in Figure 2a. Some identified genes are involved in channel activity (11 cases, six genes identified: *CACNA1A*, *KCNC2*, *KCNT1*, *SCN1A*, *SCN2A*, and *SCN3A*). Another six of the identified genes (seven cases) are implicated in vesicles trafficking, cell adhesion or transport mechanisms in general (*PCDH19*, *STXBP6*, *TBC1D24*, *DYNC1H1*, *SLC13A5*, and *ATP1A3*); four genes encode for proteins that bind DNA or RNA (four cases: *MECP2*, *PHF21A*, *SMC1A*, and *CHD2*), and three genes enabling protein binding perform various functions (four cases: *ALDH7A1*, *SHANK3*, and *ATP6V1A*). Among the other genes, *MED13L*, *WWOX*, and *TSC2* are involved in the mechanisms of gene expression (transcription), two genes (*GLB1*, and *DHRS9*) are involved in the metabolic process, and finally, the other three cases are relative to genes involved in the metabolism of protein (*PIGN*, *QARS*). Three new candidate genes for DEEs were identified in the present study: *KCNC2*, *STXBP6*, and *DHRS9*.

The presence of possible CNVs was excluded by software analysis of the homozygosity of the regions (Ion Reporter™ - version 5.18.0.22 - Software Copy Number Variation Analysis); however, all unresolved cases were sent through molecular cytogenetics at a later stage in order to obtain a more specific and sensitive CNV analysis.

The most frequently identified type of inheritance was autosomal dominant de novo (60% of cases) followed by autosomal recessive in homozygosity (17%) and heterozygosity (11%), autosomal dominant inherited from parental mosaicism (6%) and X-linked dominant de novo (6%). The inheritance of variants identified by exome sequencing is depicted in Figure 2b.

The most frequent mutations identified were missense in about two-thirds of cases (75%), followed by frameshift deletions (16%) and frameshift duplications (5%), and, finally, splicing mutations (3%)—see Figure 2c.

### 2.3. New Candidate Genes

Three new candidate genes for DEEs were identified in the present study.

**Figure 3:**
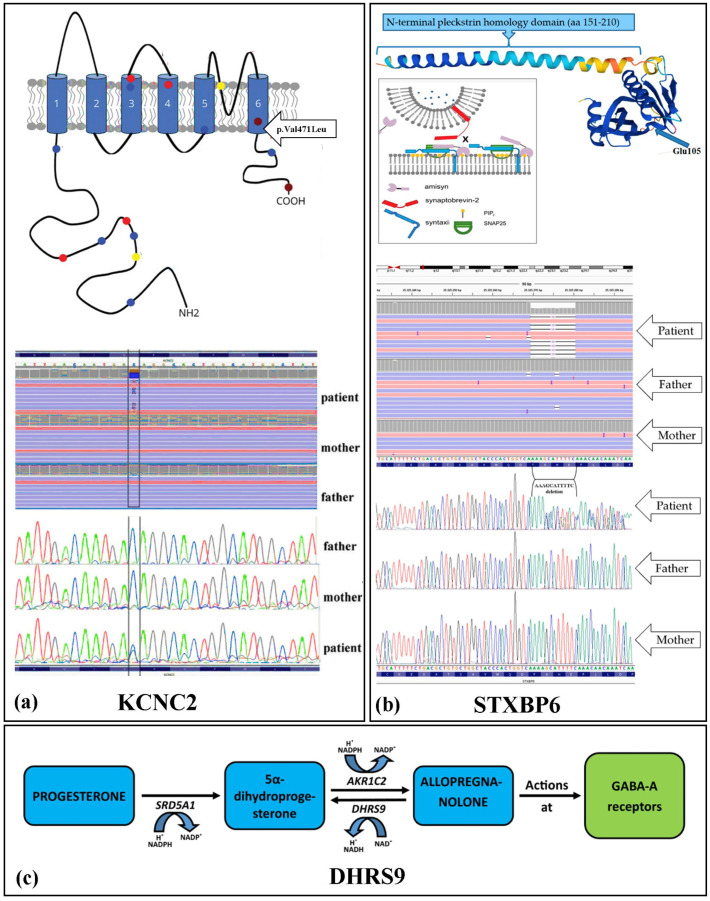
(**a**) Top: schematic structure of the KV 3.2 subunit (*KCNC2* Gene). The subunit consists of 6 Ttansmembrane segments. The missense variant (P.Val471Leu) (arrow) in the latter transmembrane segment results in a malfunctioning Protein (Modified by [18]). Down: Next-generation sequencing (NGS) and Sanger sequencing displaying de novo heterozygous missense mutation (modified from [15]). Variant allele fraction in the IGV (Integrative Genomics Viewer) images: Patient’s reads total = 84. The numbers of reads that map to either the reference or the mutated allele at the heterozygous variation of the *KCNC2* gene are, respectively, 45 (reads “C”) and 39 (reads “G”). (**b**) Top: Model of the role of amisyn in secretory vesicle exocytosis (modified from [19]). Down: Next-generation sequencing (NGS) and Sanger sequencing displaying de novo heterozygous deletion results in a frameshift and premature termination codon (p.Glu105Ter) in the patient. Next-generation sequencing (NGS) visualization with the IGV of the deletions of GAAAATGCTTT nucleotides (reverse) in the *STXBP6* gene. Reads total: 123 (AAAGCATTTTC = 48 reads; deletion = 54 reads) (modified from [17]). (**c**) Biosynthetic pathway of the allopregnanolone from progesterone. The patient shows a compound heterozygote for two mutations in the *DHRS9* gene (modified from [16]).

The *KCNC2* (potassium channel, voltage-gated, shaw-related subfamily, member 2—OMIM# 176256) gene, located at position 12q21.1, encodes for the voltage-dependent potassium subunit Kv3.2. Our exome sequencing performed in a patient with DEE, spastic quadriplegia and opisthotonos attacks revealed a de novo heterozygous variant c.1411G>C (p.Val471Leu) in the *KCNC2* gene [15]. The change in the p.Val471Leu amino acid occurred at the level of the S6 transmembrane domain in the Kv3.2 protein; this domain is involved in the formation of the pore domain of the potassium channel (Figure 3a). The results of in silico prediction of the effects of the missense de novo variant are shown in Supplementary Table S1.

DHRS9 (short-chain dehydrogenase/reductase family, member 9—OMIM# 612131) is a gene located on 2q31.1, which encodes for the NAD-dependent enzyme 3 alpha-hydroxysteroid dehydrogenase, causing the back-oxidation of 3β-tetrahydroprogesterone, also known as allopregnanolone, to 5α-dihydroprogesterone. Allopregnanolone is an allosteric modulator of GABA due to its action on GABA-A receptors. Through WES, we showed compound heterozygosity for two missense mutations, c.785C>T (p.Ser262Leu) and c.1036G>C (p.Asp346His), inherited from the mother and father, respectively. The two mutations cause a change in two highly conserved amino acid residues in the protein [16]. The results of the in silico prediction of the effects of the missense de novo variant are shown in Supplementary Table S2.

*STXBP6* (syntaxin-binding protein 6—OMIM #607958) is a gene coding for the protein amisyn, which is capable of binding components of the SNARE complex by regulating the fusion of synaptic vesicles with the cell membrane and thus playing a role in neurotransmitter homeostasis. Our exome sequencing showed a de novo c.313_323delGAAAATGCTTT variant in the *STXBP6* gene (NM_014178.8) [17]. This de novo deletion resulted in a premature stop codon at p.Glu105Ter resulting in a truncated protein (105 versus 210 amino acids). The truncated region falls in the homology domain at the N-terminal pleckstrin (such domains are usually involved in cell signaling and allow the binding of charged phosphoinositide head groups, ensuring binding to membrane lipids). This mutation results in the absence of the syntaxin-binding protein domain; therefore, the truncated protein cannot exert its regulatory and inhibitory action over the SNARE complex, and the reflexive instigation of synaptic vesicle release (Figure 3b).

## 3. Discussion

The present study aimed at analyzing the results of the use of WES as a first-line molecular genetic test in a sample of DEEs characterized by early-onset drug-resistant epilepsy and impaired cognitive development. This is the first study in the literature analyzing the diagnostic role of WES in DEEs in the Italian population; worldwide, there is only one similar study that was published in the European Journal of Human Genetics in 2023 by Boonsimma et al. [20]. In that study, 103 patients with drug-resistant early-onset epilepsy underwent exome sequencing as a first-line genetic diagnostic test. The examination revealed pathogenic or probably pathogenic variants in 62% of cases (64/103), and in 29% of cases (19/66) the variants had never been described before [20]. The difference in the detection rate—43% (35/82) in our study versus 62% (64/103) in the study of Boonsimma et al.—can be reduced to the more restrictive inclusion criteria used, which considered only epilepsies with onset in the first year of life, thus maximizing and likely selecting epilepsies with a higher probability of genetic etiopathogenesis. In terms of diagnostic yield, the results obtained from our study are solidly comparable to those obtained from large studies using WES in the diagnosis of genetic epilepsies and showing detection rates ranging from 25 to 45% [21,22]. Another very interesting difference between the two studies is the types of variants identified. In fact, the present study identified recessive variants in homozygosity in 17% of cases, while Boonsimma et al. identified a single variant in homozygosity (~1%). This significant discrepancy between the studies can be explained by the likely more conserved genetic enclave present in Sicily, which determined that identical recessive mutations are more frequent in the island population.

Similar to our study, Boonsimma et al. also found no statistically significant differences in seizure semeiology, EEG abnormalities, brain MRI findings, number of AEDs used, age, and sex between subjects with identified or unidentified variants. The only weak significance obtained (*p* = 0.048) is related to the nenonatal onset of seizures compared to onset at other ages. Such weak evidence would support a greater “genetic potential” in neonatal onset epilepsies [20].

In light of cost reductions related to exomic sequencing kits, the diagnostic protocol of using WES as the first-line test is also a cost-effective option compared with protocols involving an NGS panel of genes first. Palmer et al., in their research aimed at investigating the cost-effectiveness of using WES in the diagnostic protocol, showed that exome sequencing is about ten times cheaper than the standard diagnostic model, and the net savings per variant identified are more than EUR 3000 [23].

Another undeniable advantage of using WES is the possibility of identifying new possible candidate genes; the present study led to the identification of three genes never before related to epilepsy, namely, *KCNC2*, *STXBP6* and *DHRS9*.

The *KCNC2* gene, located at position 12q21.1, encodes for the voltage-dependent potassium subunit Kv3.2. Currents mediated by the homotetramers of Kv3.2 are defined as of the delayed-rectifier type because their conductance begins with the depolarized membrane from about −10 mV, resulting in rapid repolarization and thus shortening the duration of the action potential [24]. These properties are critical to the high-frequency firing of specific neuronal populations, in which Kv3.2 and Kv3.1 are expressed due to their ability to rapidly activate and deactivate following voltage changes [25]. Kv3.2 channels are selectively expressed in the central nervous system and, in particular, are found at high levels in populations of GABAergic inhibitory interneurons located in the cortex, hippocampus, amygdala, and caudate; they are characterized by a “fast-spiking” pattern and contain specific markers such as parvalbumin and somatostatin [26,27]. There are four isoforms of Kv3.2 (Kv3.2a-Kv3.2d) derived from alternative splicings of the *KCNC2* gene, resulting in different COOH terminal domains, which, however, appear not to alter the biophysical properties of the channel, but rather their subcellular localization [27,28].

We reported for the first time in 2020 a case of DEE, spastic quadriplegia, opisthotonos attacks, identified by a de novo variant in the *KCNC2* gene shown thanks to WES sequencing. The change in the p.Val471Leu amino acid occurred at the level of the S6 transmembrane domain in the Kv3.2 protein; this domain is involved in the formation of the pore domain of the potassium channel (Figure 3a). In addition, Val at position 471 is conserved in all members of the Kv3 subfamily of potassium channels-KCNC1, KCNC2, KCNC3 and KCNC4. Due to our first *KCNC2* signaling in DEEs [15] and subsequent reports from the international scientific community, today, the KCNC2 gene (OMIM# 176256) is considered responsible for Developmental and Epileptic Encephalopathy 103 (DEE103; 619913).

DHRS9, which encodes for the NAD-dependent enzyme 3 alpha-hydroxysteroid dehydrogenase, catalyzes the back-oxidation of 3β-tetrahydroprogesterone, also known as allopregnanolone, to 5α-dihydroprogesterone. Allopregnanolone is an allosteric modulator of GABA due to its action on GABA-A receptors. Allopregnanolone is synthesized at the central nervous system level by progesterone through the sequential action of two enzymes: 5α-reductase type I (5α-RI) (SRD5A1 gene), which converts progesterone to 5α-dihydroprogesterone, and 3α-hydroxysteroid hydrogenase (3α-HSD) (AKR1C2 gene), which converts 5α-dihydroprogesterone to allopregnanolone (3β-tetrahydroprogesterone) [29]. WES performed on a girl with early onset epilepsy revealed that she had a compound heterozygote for two novel missense mutations of the *DHRS9* gene likely to disrupt protein function. In 2020, for the first time, we reported a case of DEE related to DHRS9 gene mutation. The absence or dysfunction of *DHRS9* causes the dysregulation of the back-oxidation of 3-tetrahydroprogesterone to 5-dihydroprogesterone (Figure 3c), provoking an impairment at the allopregnanolone level with a consequent effect on inhibitory gabaergic neurons [16,30].

*STXBP6* is a gene coding for the protein amisyn that is capable of binding components of the SNARE complex. SNAREs (soluble N-ethylmaleimide-sensitive factor attachment protein receptor) are a complex family of proteins implicated in synaptic vesicle exocytosis and synaptic transmission. Amisyn has an R-SNARE motif at the C-teminal that can substitute for the synaptobrevin motif in the formation of the complex; therefore, amisyn acts as a negative regulator of the SNARE complex by inhibiting exocytosis through interference with the synaptobrevin action. All of the genes constitutive of the SNARE complex (*VAMP2*-synaptobrevin; *STX1A*-synaptobrevin1; *SPAP25* gene), except amysin, have already been described as being responsible for epilepsy, DEE, and/or neurodevelopmental disorders when mutated [31,32]. Ours is the first study to show evidence that a mutation in the *STXBP6* gene correlates with a form of DEE as a comorbidity with autism spectrum disorder [17]. The causative mutation detected by exome sequencing determined the phenotypic picture by altering the synaptic vesicle fusion process essential to their exocytosis.

Even in cases of genes that would have been identified by a traditional NGS panel, exome sequencing provides a broader view of the patient’s genetic makeup. At present, the possible additive or resilience effects of additional variants beyond the likely pathogenic one remain largely speculative. An improvement in our understanding of the role of genetic concurrences and multi-hit etiologies, as are likely underlying many neurodevelopmental disorders, together with the development of bioinformatics tools will allow us in the near future to carry out this additional diagnostic step, which involves the use of broad-spectrum sequencing techniques as a fundamental prerequisite.

In light of the evidence in favor of the use of WES as a first-line molecular genetic test, positive clinical benefits appear evident that should be considered by the national and international organizations dealing with the treatment and assistance of people with epilepsy, which until now have assigned to exome sequencing a role secondary to other methods [12].

It is important to note that both whole-exome sequencing (WES) and whole-genome sequencing (WGS) have different strengths and limitations, and their effectiveness depends on the specific use case and goals of the genetic test. WES focuses on sequencing the protein-coding regions of the genome (exons). These regions are more likely to contain disease-causing variants, so WES is particularly useful for identifying genetic variants associated with specific disorders or conditions. By sequencing a smaller portion of the genome, WES is generally faster and more cost-effective than WGS. On the other hand, WGS provides a comprehensive and detailed analysis of the entire genome, including both exons and non-coding regions, such as introns. In this latter case, single nucleotide variations can be responsible for long-range genomic effects, leading to alterations in the expression pattern of contiguous genes, as described, for example, by the SNP rs12913832 located in intron 86 of the *HERC2* gene, an intronic variant involved in the expression of the adjacent *OCA2* gene [33,34], which plays a role in human pigmentation. This allows for a more comprehensive understanding of an individual’s genetic makeup, including variants that may be found in non-coding regions and regulatory elements. WGS can be particularly helpful in research settings, as it provides a broader view of the genome and allows for the discovery of novel genetic associations. In summary, both WES and WGS have their own advantages and are appropriate for different purposes. The choice between the two depends on the specific needs and objectives of the genetic test. However, as reported by Ostrander et al., 2018 [35], the use of whole-genome analysis (WGA) efficiently reveals the different variants, and, in future, may be used as an efficient strategy for the clinical diagnosis of all genetic conditions.

The finding of genetic mutations in our study that are also present in other patients (c.433C>T MECP2 and c.1025C>T SCN1A) (Table 3) refers to hotspot regions and represents an important step forward in understanding the basis of genetic diseases. In total, 141 and 47 publications, respectively, are related to these variants (Varsome). The fact that these mutations were found in several patients lends greater validity to the results of our analysis, and provides an element of confirmation that these mutations play an important role in the pathogenesis of the disease. Furthermore, the finding of mutations in hotspot regions is an important research finding that has significant implications for understanding genetic diseases, developing targeted therapies, and deepening our knowledge of human evolution.

Although the present study has obtained important scientifically relevant results, it has some limitations. First of all, sample size was limited by the COVID-19 pandemic and the rarity of the disease under study.

The use of WES has the merit of being able to detect mutations in genes previously unrelated to epilepsy; however, it must be considered that, according to ACGM criteria, very strong evidence of pathogenicity occurs only in relation to already known genes, in which the causative mechanism of pathology is largely explicit. Therefore, sometimes, if variants in genes not previously related to the pathology under study are identified, they are to be considered as probably pathogenic, pending new scientific evidence.

Another limitation related to the newly identified possible candidate genes is that it is impossible to outline an accurate genotype–phenotype correlation with a single case being examined, in light of the high phenotypic variability that characterizes DEEs. It should, however, be emphasized that it is also important to report single cases, especially in genes never before described, in order to entice the scientific community to collect similar cases and develop a more accurate genotype–phenotype correlation.

Finally, WES results in the identification of numerous variants of uncertain significance. With current methods, it is extremely difficult to understand what role such variants play within the phenotype observed in the patient.

## 4. Materials and Methods

### 4.1. Patient Selection

WES was performed on patients referred to the Oasi Research Institute—IRCCS in Troina from 2019 to 2023. Seven exome sequencings had previously been performed and were included in the study as they met the inclusion and exclusion criteria.

All patients met the following inclusion and exclusion criteria.

Inclusion criteria: history of focal or generalized drug-resistant seizures with onset within 36 months of age associated with global developmental delay and/or intellectual disability; DNA available from both parents; MRI and EEG data available for the participating subject; written informed consent form provided by participants. All enrolled subjects were evaluated by a child neurologist/neuropsychiatrist and a geneticist at least once.

Epilepsy was considered drug-resistant if it met the ILAE definition of drug resistance, i.e., “failure of adequate trials of two tolerated, appropriately chosen and used antiepileptic drug schedules (whether as monotherapies or in combination) to achieve sustained seizure freedom” [36].

Exclusion criteria: patients presenting with multi-organ systemic dysfunctions; presence of formerly known genetic diagnoses and/or known micro-rearrangements identified by traditional and high-resolution molecular cytogenetic (CGH-array) methods.

Patients with multi-organ systemic dysfunctions and dysmorphisms suggestive of the possible known syndromic picture were excluded from the study and referred for conventional and molecular cytogenetic investigation.

All patients underwent thorough clinical and medical personal and family history evaluations, thorough neurological examinations, neuropsychological testing at the discretion of the psychological team according to the clinical features of the case, EEG recordings, and neuroimaging study by magnetic resonance imaging (MRI).

The study was approved by the Ethics Committee of the Oasis Research Institute-IRCCS of Troina (Protocol CE/37 as of 3 June 2017, approval code: 2017/05/31/CE-IRCCS-OASI/9). Written informed consent was obtained from the patients’ parents. This clinical trial was conducted in accordance with all protocol requirements and according to the Good Clinical Practice Guidelines and the principles of the Declaration of Helsinki.

### 4.2. Exome Sequencing and Variant Interpretation

Genomic DNA was isolated from peripheral blood leukocytes. Whole-exome sequencing (WES) analysis was performed at the Oasi Research Institute—IRCCS, Troina, Italy, using libraries designed using the AmpliSeqTM Exome technology (Thermo Fisher Scientific, Foster City, CA, USA). In total, 100 ng of gDNA was used as the starting material. The extraction protocol applied was a non-organic and non-enzymatic extraction method, as previously described [37]. We used pooled libraries to emulsify PCR on the Ion CHEF instrument according to the manufacturer’s protocol (Thermo Fisher Scientific). In total, 97% and 95% of regions of interest (ROI) had a minimum coverage of at least 20× and 30×, respectively. All libraries were diluted to 100 pM and then loaded into the Ion CHEF (Thermo Fisher Scientific), following standard protocols. The generated amplicons were genotyped with the IonS5plus platform, following the instructions of the Thermo Fisher Scientific protocols. Base-calling and sequence alignment were performed for all samples (TRIOS), using the Ion Torrent Suite v.5 software, and genetic variants were identified, using the Variant Caller of Torrent Suite v.5 with optimized parameters provided by the manufacturer for the AmpliSeq Exome. Sequences were aligned with the GRCh37/hg19 reference genome using the TMAP alignment (Thermo Fisher Scientific). WES data processing was performed using BAM, BAI and FASTQ files, as well as detected variant files (VCF and TSV), using IonReporter software v. 5.18.0.22 (Thermo Fisher Scientific, Foster City, CA, USA) and/or wANNOVAR [38]. DNA sequences were displayed using Integrated Genomics Viewer [39]. The information obtained by “CoverageAnalysis” was used to measure the average depth and percent coverage of each gene. Analytical sensitivity of the assay: the NGS assay is able to reveal substitution variants in the analyzed sequence (exons and splicing sites) and is unable to detect deletions/duplications. Variants with “minor allele frequency” (MAF) greater than 1% found in the databases of the 1000 Genomes Project and 6500 exome project are considered as population polymorphisms, and were not considered as playing a possible pathogenic role.

All resulting variants were compared with the following polymorphism/mutation databases: ExAC (http://exac.broadinstitute.org/), ESP (http://evs.gs.washington.edu/EVS/), HGMD (http://www.hgmd.cf.ac.uk), and GnomAD (https://gnomad.broadinstitute.org/) accessed on 8 nov 2023. The pathogenicity of the Missense variants was evaluated, using the following in silico softwares: SIFT (version 6.2.1), PolyPhen-2 (version 2.2.3), MutationTaster (version 2021), CADD (version 1.6). In addition, nucleotide conservation was evaluated using the PhastCons and PhyloP programs. However, standard procedures were used for the assessment of the pathogenicity of variants (ACMG criteria) [40], categorized as pathogenic, likely pathogenic, uncertain, likely benign and benign. VarSome was used to allow fast and accurate variant discovery, as well as the annotation and interpretation of NGS data. VarSome enables variant classification according to the guidelines of the ACMG: The American College of Medical Genetics and Genomics (ACMG) recommends five variant classification categories (pathogenic, likely pathogenic, uncertain significance, likely benign, and benign), and these have been widely used in genetics studies. Variant interpretation and classification was performed following the Guidelines of the American College of Medical Genetics (ACMG) [41].

Software algorithms (Ion Reporter™ Software Copy Number Variation Analysis, version 5.18.0.22) were used to search all samples for possible CNVs [42].

Sanger sequencing was performed to confirm the putative variants obtained after genotyping WES analysis. Finally, segregation analysis of the identified variant was performed using parents and/or relatives.

### 4.3. Statistical Analysis

The Fisher exact test was used to analyze a possible correlation between demographic and clinical characteristics (sex, time of onset of epilepsy, family history of epilepsy, type of EEG abnormalities, presence of MRI abnormalities) and the presence of causative variants identified by WES. Statistical analysis was performed using SPSS Statistics version 26. A *p*-value less than 0.05 was considered statistically significant.

## 5. Conclusions and Future Prospects

The present study investigated the efficacy of using exome sequencing as a first-line molecular genetic test in a sample of subjects with DEEs admitted to the Oasi Research Institute—IRCCS in Troina (Italy).

Subjects with early-onset epilepsy with associated global developmental delay, developmental disorders, and, later, intellectual disability, as well as a rather severe clinical phenotype, are often included in DEEs and frequently identify a genetic etiopathogenesis. The phenotypic spectrum of such forms is extremely broad and includes a multitude of neurological signs, which are invariably associated with psychiatric manifestations, and psychological and behavioral abnormalities. Such a symptomatology reflects the wide genotypic variability underlying the various syndromes and adds to the different resilience and vulnerability factors, which both genetic and environmental factors determine. In this context, it is of extreme importance to have available diagnostic tools that, with a good cost-effectiveness ratio and within a reasonable time frame, can shed light on the etiological nature of the encephalopathy.

The main result obtained from the global analysis of the 82 WES performed is a detection rate of 43% (35 out of 82), a remarkable result that adheres to the average of studies found in the literature on patient populations similar to ours.

This experimental study, although with the limitations indicated in the previous paragraph, has shown that WES constitutes an effective first-line diagnostic technique in the precise diagnosis of epilepsies with likely genetic etiology, thanks, above all, to its ability to sequence large portions of human DNA, which results in a high detection rate. Furthermore, it has been shown that although several genes responsible for the most frequent DEEs have been identified in recent decades, there is still room to identify new genes related to epilepsy.

These findings represent a foundation for further future developments in this area of research. The contributions made here to the international scientific community are crucial in terms of actively reporting other individuals with mutations in the new candidate genes identified in this study, in order to make a more precise genotype–phenotype correlation so to determine the broadness of the phenotypic spectrum of these new forms of DEEs.

Many studies also suggest that long-read sequencing is an effective additional tool for the molecular diagnosis of genetic disorders in patients unresponsive to conventional technologies [43,44,45,46]. We believe that, in epilepsy, the diagnostic tool with the most potential will be multi-omics.

Finally, the determination of an accurate genetic diagnosis is the fundamental prerequisite of precision therapy. Precision medicine, that is, the attempt to personalize prevention, diagnosis, and treatment as much as possible according to the characteristics and needs of the individual patient, must be a primary goal of clinical research and the new mandate of modern medicine, which replaces obsolete categorical, massifying, and nosographic approaches with new, more humane and more effective practices that recognize each patient’s uniqueness.

## Figures and Tables

**Table 1:** Main features of the 82 subjects with DEE participating in the study.

Features	No.	Patients with Causing Variant Found	*p*-Value
Total	82	35 (43%)	
Males	40 (49%)	15 (43%)	NS
Females	42 (51%)	20 (57%)	
Age in years (±SD)	10 (6)	9 (6)	
Epilepsy onset in months (SD)	16 (13)	15 (14)	
Neonatal	17 (21%)	10 (29%)	NS
Infant	65 (79%)	25 (71%)	
Familiarity with epilepsy	30 (37%)	12 (34%)	NS
EEG abnormalities			
Focal abnormalities	36 (44%)	16 (46%)	NS
Multifocal abnormalities	14 (17%)	6 (17%)	
Generalized abnormalities	15 (18%)	6 (17%)	NS
Burst suppression	7 (8%)	3 (9%)	
Slowing	12 (15%)	5 (14%)	
Hypsarrhythmia	4 (5%)	2 (6%)	
MRI findings			
Normal	43 (52%)	13 (37%)	NS
Cerebral atrophy	24 (29%)	9 (25%)	
Ventricular dilatation	23 (28%)	9 (25%)	
Thinning/agenesis corpus callosum	19 (23%)	7 (20%)	
Periventricular hyperintensity	9 (11%)	4 (11%)	
Other	12 (15%)	4 (11%)	
N. AEDs used before the test			
2	15 (18%)	6 (17%)	
3	31 (38%)	13 (37%)	
4	20 (24%)	9 (26%)	
≥5	16 (19%)	7 (20%)	
WES			
Trio	72 (88%)	32 (91%)	
Quadro (two patients per test)	5 (12%)	3 (9%)	
Genes identified	27		
Recurrent genes (excluding *quadro*)	5 (18.5%)		
Pathogenic mutations	34		
Recurrent mutations (two genes)	2 (5.9%)		

SD: standard deviation; EEG: electroencephalogram; MRI: magnetic resonance imaging; AEDs: antiepileptic drugs; WES: whole-exome sequencing; NS: not significant (*p* value > 0.05).

**Table 2:** Characteristics of patients with the disease-causing variant identified.

ID	Age at Testing	Sex	Clinical Features	Seizure Semiology	EEG Abnormalities	AEDs	MRI
#001	11	M	Severe ID; sleep disorder	GTCS	Focal	VPA; CLB	CA, DV
#002	8	F	Mild ID	Typical absence	Focal	VPA; ESM	Normal
#003	11	F	Profound ID; microcephaly, tetraparesis	GTCS	Multifocal; slowings	LVT, PHB, TPM, CLB, LTG, PHT	CA, DV
#004	5	F	Profound ID; behavioral disorder; microcephaly	Focal	Focal	VPA, LTG, CLB	Normal
#005	14	F	DI, microcephaly, cataract	epileptic spasm	Focal	VPA, GVG	ACC
#006	5	F	Mild ID	Myoclonic, seizures in hyperpyrexia	Generalized	VPA, CLB	Normal
#007	7	M	Severe ID, limb hypertonia,	epileptic spasm, GTCS	Focal; slowings	PH, LVT, PHT, GVG	DV, TCC, left hippocampal malrotation
#008	11	M	Severe ID	Focal, Generalized, Myoclonic	Focal	LVT, VPA, PHB	CA
#009	7	M	Severe DI; tetraparesis	Focal, Generalized, Myoclonic	Focal; slowings	VPA, CBZ, PHB	DV
#010	10	F	Severe ID; behavioral disorder	Myoclonic-Absences, GTCS	Focal	BRV, LCM, CLB, TPM	CA, DV
#011	3	M	UIDD; behavioral disorder	Myoclonic-Absences, GTCS	Generalized	TPM	CA, DV
#012	7	M	Mild ID, behavioral disorder	Focal	Focal	TGB	CA
#013	7	F	Mild ID, behavioral disorder	Focal	Focal	VPA; LVT	Normal
#014	12	F	Mild ID	Myoclonic; generalized	Diffuse	VPA, NZP, TPM	CA
#015	3	F	GDD	Focal, GTCS	Multifocal	LVT, PDX	Normal
#016	13	F	Severe ID, microcephaly, hyposomy	Focal, GTCS	Diffuse	VPA, RFM	DM; TCC
#017	5	F	Severe ID	Focal, Myoclonic, GTCS	Focal	TPM, NTP	ACC
#018	41	F	Moderate ID	Focal, GTCS in hyperpyrexia	Diffuse	LCM, PHB, CLN	ACC
#019	13	M	Severe ID, Lower limbs heterometry	Focal	Focal	CBZ, LVT	PVWMH
#020	10	F	Severe ID	Focal, GTCS	Focal	VPA, CBZ	Normal
#021	9	M	Mild ID	Focal, GTCS	Multifocal	VPA, PHT, LEV	Epidermoid cyst
#022	11	F	Profound ID; behavioral disorder	Focal	Focal	VPA, PHT	Normal
#023	3	F	Profound ID	Focal, GTCS	Multifocal; slowings	PHB, LVT, CNZ	Cerebellar atrophy
#024	5	M	Severe ID	Myoclonic, focal, GTCS	Focal	PHB, VPA	Normal
#025	5	F	Moderate ID, Ataxia	Myoclonic-Absences	Focal	LVT, ESM	PVWMH
#026	3	F	GDD	GTCS	Focal; slowings	LVT, VPA, CLB	Normal
#027	7	F	Profound ID, ASD	Epileptic spasm, Tonic.	hypsarrhythmia; Focal;	VPA, LVT, CNZ	DV
#028	13	M	Mild ID	Generalized tonic seizures, GTCS	Multifocal	OXC, RFM, VPA	Normal
#029	8	M	Profound ID, Spastic tetraparesis	epileptic spasm, Generalized tonic seizures	Burst-suppression	LVT, CLB	CA, DV, PVWMH
#030	4	F	Mild ID, behavioral disorder	Epileptic spasm, Focal	Burst-suppression	VPA, CLN	TCC, DV
#031	7	F	Severe ID, ASD, microcephaly	GTCS	Focal	LVT, VPA	simplified gyral pattern, DV, ACC
#032	6	M	UIDD, GM1 gangliosidosis, microcephaly	GTCS	Focali	CBZ, NTP	Normal
#033	10	M	Mild ID	GTCS	burst-suppresion	LVT, PHB	Normal
#034	10	M	Profound ID	Generalized tonic seizures, GTCS	Multifocal	CBZ, CLB	PVWMH
#035	11	M	Mild ID	GTCS	Multifocal	LVT, VPA	Normal

ID: intellectual disability; UIDD: unspecified intellectual developmental disorder; GDD: global developmental delay; GTCS: generalized tonic-clonic seizure; ASD: Autism Spectrum Disorder; AEDs: antiepileptic drugs: VPA: valproic acid; CLB: clobazam; LVT: levetiracetam; ESM: etosuccimide; PHB: phenobarbital; CBZ: carbamazepine; LTG: lamotrigine; PHT: phenytoin; GVG: vigabatrin; BRV: brivaracetam; LCM: lacosamide; TPM: topiramate; RFM: rufinamide; TGB: tiagabine; PDX: pyridoxine; NTP: nitrazepam; CLN: clonazepam; OXC: oxcarbazepine; CA: cortical atrophy; VD: ventricular dilatation; ACC: agenesis of corpus callosum; TCC: thinning of corpus callosum; DM: delayed myelination; PVWMH: periventricular white matter hyperintensity.

**Table 3:** Mutations with pathogenic role identified by WES.

ID	Gene	Accession Number	Nucleotide Change	Affected Protein	Variant Type	Inheritance	ACMG Criteria
#001	*CACNA1A*	NM_001127221	c.2140G>A	p.Val714Met	Missense	De novo (AD)	Pathogenic
#002	*CACNA1A*	NM_001127221	c.2667del	p.Ala890ProfsTer3	Deletion	De novo (AD)	Pathogenic
#003	*QARS*	NM_005051	c.134G>T	p.Gly45Val	Missense	Homozygous (AR)	Pathogenic
#004	*SHANK3*	NM_001372044.2	c.4044_4045del	p.Pro1349CysfsTer8	Deletion	De novo (AD)	Pathogenic
#005	*DYNC1H1*	NM_001376.5	c.4868G>A	p.Arg1623Gln	Missense	De novo (AD)	Pathogenic
#006	*SCN1A*	NM_001165963	c.1025C>T	p.Ala342Val	Missense	De novo (AD)	Pathogenic
#007	*WWOX*	NM_016373.4	c.1043del	p.Ala1558Serfs*6	Deletion	Homozygous (AR)	Pathogenic
#008 *	*SLC13A5*	NM_177550.5	c.1280C>T	p.Ser427Leu	Missense	Homozygous (AR)	Pathogenic
#009 *	*SLC13A5*	NM_177550.5	c.1280C>T	p.Ser427Leu	Missense	Homozygous (AR)	Pathogenic
#010 *	*PIGN*	NM_176787.5	c.1694G>T	p.Arg565Leu	Missense	Homozygous (AR)	Likely Pathogenic
#011 *	*PIGN*	NM_176787.5	c.1694G>T	p.Arg565Leu	Missense	Homozygous (AR)	Likely Pathogenic
#012 *	*KCNT1*	NM_020822.2	c.862G>A	p.Gly288Ser	Missense	Parental mosaicism (AD)	Pathogenic
#013 *	*KCNT1*	NM_020822.2	c.862G>A	p.Gly288Ser	Missense	Parental mosaicism (AD)	Pathogenic
#014	*CHD2*	NM_001271.3	c.2663A>G	p.Asp888Gly	Missense	De novo (AD)	Pathogenic
#015	*ALDH7A1*	NM_001201377.1	c.1208C>T c.435A>T	p.Pro403Leu p.Arg145Ser	Missense Missense	Compound heterozygous (AR)	Pathogenic Likely Pathogenic
#016	*SMC1A*	NM_006306.3	c.611_612del	p.Glu204GlyfsTer3	Deletion	De novo (XLD)	Pathogenic
#017	*MECP2*	NM_001110792.1	c.433C>T	p.Arg145Cys	Missense	De novo (XLD)	Pathogenic
#018	*SCN1A*	NM_001165963	c.1025C>T	p.Ala342Val	Missense	De novo (AD)	Pathogenic
#019	*ATP6V1A*	NM_001690	c.944C>T	p.Thr315Ile	Missense	De novo (AD)	Pathogenic
#020	*TSC2*	NM_000548	c.4678G>A	p.Ala1560Thr	Missense	De novo (AD)	Likely Pathogenic
#021	*SCN3A*	NM_006922	c.3070G>A	p.Glu1024Lys	Missense	De novo (AD)	Likely Pathogenic
#022	*SHANK3*	NM_001127221	c.6660_6661Dup	p.Pro2221LeufsTer287	Insertion	De novo (AD)	Pathogenic
#023	*ATP1A3*	NM_152296	c.2116G>C c.1756C>G	p.Gly706Arg p.Arg586Gly	Missense Missense	Compound heterozygous (AR)	Pathogenic Likely Pathogenic
#024	*TBC1D24*	NM_001199107.1	c.457G>A	p.Glu153Lys	Missense	De novo (AD)	Likely Pathogenic
#025	*MECP2*	NM_001110792.1	c.433C>T	p.Arg145Cys	Missense	De novo (AD)	Pathogenic
#026	*PCDH19*	NM_001184880.2	c.1019A>G	p.Asn340Ser	Missense	De novo (AD)	Pathogenic
#027	*SCN2A*	NM_001040143.1	c.719C>T	p.Ala240Val	Missense	De novo (AD)	Pathogenic
#028	*GABRB3*	NM_000814.6	c.911A>G	p.Lys304Arg	Missense	De novo (AD)	Pathogenic
#029	*KCNC2*	NM_139137.3	c.1411G>C	p.Val471Leu	Missense	De novo (AD)	Pathogenic
#030	*DHRS9*	NM_001289763	c.785C>T c.1036G>C	p.Ser262Leu p.Asp346His	Missense Missense	Compound heterozygous (AR)	Likely Pathogenic Likely Pathogenic
#031	*STXBP6*	NM_014178.8	c.313_323del	p.Glu105Ter	Deletion	De novo (AD)	Likely Pathogenic
#032	*GLB1*	NM_000404	c.1480-2A>G c.1769G>A	New protein p.Arg590His	Splicing Missense	Compound heterozygous (AR)	Pathogenic Pathogenic
#033	*PHF21A*	NM_001101802	c.649_650del	p.Gln217ValfsTer6	Deletion	De novo (AD)	Pathogenic
#034	*SCN2A*	NM_021007	c.2387T>C	p.Leu796Pro	Missense	De novo (AD)	Pathogenic
#035	*MED13L*	NM_015335	c.4670dup	p.Ala1558SerfsTer6	Insertion	De novo (AD)	Pathogenic

AD: autosomal dominant; AR: autosomal recessive; XLD: X-linked dominant. * Two patients per family: see Table 1.

## Data Availability

The data presented in this study are available on request from the corresponding authors.

## Supplementary Materials

The following supporting information can be downloaded at: https://www.mdpi.com/article/10.3390/ijms25021146/s1.
