# Supplementary material for: Whole Exome Sequencing as a First-Line Molecular Genetic Test in Developmental and Epileptic Encephalopathies
Source: Int J Mol Sci. 2024 Jan 17;25(2):1146. doi: 10.3390/ijms25021146 (PMC10816140; doi:10.3390/ijms25021146)
Supplement: Supplementary file 1 [file ijms-25-01146-s001.zip › Supplementary Table-S1.pdf]

Table S1: *In silico* prediction of the effects of missense *de novo* variant c.1411G>C (p.Val471Leu) in the *KCNC2* (KV3.2) gene.

In silico predictive tool	***Prediction/Score/PHRED-scaled***
PA_CADD_phred	27.3 (>30 highly pathogenic; >20 pathogenic)
PA_DANN_score	0.998239 (range from 0 to 1) *
PA_FATHMM_pred	DAMAGING
PA_GERP++_RS	6.06 (rangefrom 12.3 to 6.17) *
PA_LRT_pred	Deleterious
PA_M-CAP_pred	DAMAGING
PA_MetaSVM_pred	DAMAGING
PA_MutationAssessor_pred	Low (non-functional)
PA_MutationTaster_pred	DAMAGING
PA_PROVEAN_pred	DAMAGING
PA_Polyphen2_HDIV_pred	Probably Damaging
PA_SIFT_pred	DAMAGING
PA_SiPhy_29way_logOdds	20.6397 (range from 0 to 37.9718) *
PA_fathmm-MKL_coding_pred	DAMAGING

*A larger number indicates a higher probability to be damaging. Modified from ref. [15].
