# Supplementary material for: Whole Exome Sequencing as a First-Line Molecular Genetic Test in Developmental and Epileptic Encephalopathies
Source: Int J Mol Sci. 2024 Jan 17;25(2):1146. doi: 10.3390/ijms25021146 (PMC10816140; doi:10.3390/ijms25021146)
Supplement: Supplementary file 1 [file ijms-25-01146-s001.zip › Supplementary Table-S2.pdf]

Table S2: In silico prediction of the effects of missense mutations, c.785C > T (p.Ser262Leu) and c.1036G > C (p.Asp346His) in the DHRS9 (NM_001289763) gene.

In Silico Predictive Tool	***Prediction/Score***		***PHRED-Scaled***
Mutation	***c.785C > T (p.Ser262Leu)***	***c.1036G > C (p.Asp346His)***	
dbSNP	rs776765324	rs11695788	
PA_CADD_phred	21.5	29.6	>30 highly pathogenic; >20 pathogenic
PA_DANN_score	0.996382	0.995699	range from 0 to 1 *
PA_Eigen-phred	1.87611	13.87712	
PA_FATHMM_pred	DAMAGING	DAMAGING	
PA_GERP++_RS	5.14	5.93	range from 12.3 to 6.17 *
PA_LRT_pred	Deleterious	Deleterious	
PA_M-CAP_pred	DAMAGING	DAMAGING	
PA_MetaSVM_pred	DAMAGING	DAMAGING	
PA_MutationAssessor_pred	MEDIUM	HIGHT	neutral, low, medium, high
PA_MutationTaster_pred	Polymorphism	DAMAGING	
PA_PROVEAN_pred	DAMAGING	DAMAGING	
PA_Polyphen2_HDIV_pred	Benign	Probably Damaging	
PA_SIFT_pred	Tolerated	Deleterious	
PA_SiPhy_29way_logOdds	16.9774	19.9359	range from 0 to 37.9718 *
PA_fathmm-MKL_coding_pred	DAMAGING		
ClinVar (Clinical Significance)	Not Reported in ClinVar	Not Reported in ClinVar	
Frequency (GnomAD)	0.000004	0.003797	

* A larger number indicates a higher probability to be damaging. Modified from ref. [16].
